# Supplementary material for: CHIT1 at diagnosis predicts faster disability progression and reflects early microglial activation in multiple sclerosis
Source: Nat Commun. 2024 Jun 12;15:5013. doi: 10.1038/s41467-024-49312-y (PMC11169395; doi:10.1038/s41467-024-49312-y)
Supplement: Supplementary file 1 — Supplementary Information [file 41467_2024_49312_MOESM1_ESM.pdf]

CHIT1 at diagnosis predicts faster disability progression and reflects early microglial activation in multiple sclerosis

Supplementary Table 1. Protein measurements for CSF biomarkers and quality control.

	n measured	Inter-plate CV (%)	Intra-plate CV (%)	n CV > 20%	n < or > detection range	n passed QC (%)
**CHIT1 (1)**	140	6.6%	5.9%	5	6 < and 1 >	129 (92.1%)
**CHIT1 (2)**	56	1.9%	8.0%	4	0 < and 2 >	51 (91.1%)
**CHI3L1**	143	17.7%	3.4%	0	1 < and 1 >	141 (98.6%)
**sTREM2**	140	10.1%	7.1%	7	20 < and 0 >	118 (80.7%)
**GPNMB**	196	11.8%	3.0%	0	1 < and 0 >	195 (99.5%)
**CCL18**	196	17.3%	5.7%	2	4 < and 0 >	190 (96.9%)
**NfL (1)**	143	12.3%	13.5%	15	1 < and 6 >	122 (85.3%)
**NfL (2)**	55	0.78%	2.4%	0	0 < and 2 >	53 (96.4%)

(1) refers to protein measurements for these CSF biomarkers already included in previous work from our group[1]. (2) refers to the additional measurements incorporated in the current study. CV, coefficient of variation; QC = quality control.

Supplementary Table 2. Correlation matrix of CSF biomarkers.

	**CHIT1** (n = 180)	**CHI3L1** (n = 135)	**sTREM2** (n = 118)	**GPNMB** (n = 192)	**CCL18** (n = 187)	**NfL** (n = 169)
**Median (IQR)**	3.6 (3.2 – 4.0)	2.3 (2.1 - 2.5)	3.4 (3.2 - 3.5)	3.8 (3.6 - 3.9)	2.5 (2.4 - 2.7)	3.0 (2.8 - 3.4)
**CHIT1**		128	113	180	175	158
**CHI3L1**	0.48***		118	135	132	113
**sTREM2**	0.00	0.14		118	115	98
**GPNMB**	0.41***	0.46***	0.17		187	169
**CCL18**	0.10	0.28**	0.17	0.22**		164
**NfL**	0.31***	0.15	-0.10	0.01	-0.03	

The left-bottom side of the matrix contains the Pearson correlation coefficients (r). The right-top side of the matrix contains the number of measurements within each correlation. P-values (*P*) are derived from two-sided Pearson correlation test, with *$P \leq 0.05$, **$P \leq 0.01$, ***$P \leq 0.001$ (exact P-values: CHIT1-CHI3L1, <0.0001; CHIT1-GPNMB, <0.0001; CHIT1-NfL, <0.0001; CHI3L1-GPNMB, <0.0001; CHI3L1-CCL18, 0.0013; GPNMB-CCL18, 0.0022). The first row includes the median and interquartile range (IQR) CSF concentrations for each biomarker. CHIT1 (log, pg/ml), CHI3L1 (log, ng/ml), sTREM2 (log, pg/ml), GPNMB (log, pg/ml), CCL18 (log, pg/ml) and NfL (log, pg/ml). n = number of measurements included in the correlation analysis.

Supplementary Table 3. Linear regression of CSF biomarkers with CSF hallmarks.

Variable (Outcome)	CHIT1 (n = 180)		CHI3L1 (n = 135)		sTREM2 (n = 118)		GPNMB (n = 192)		CCL18 (n = 187)	
	**β**	***P***	**β**	***P***	**β**	***P***	**β**	***P***	**β**	***P***
IgG index	0.36	**0.013**	1.10	**0.0015**	0.65	0.036	0.85	**0.0032**	-0.63	**0.038**
OCB count	0.35	**0.0083**	1.21	**0.00010**	0.47	0.074	0.91	**0.0015**	-0.48	0.12
WBC count	0.03	0.87	0.81	0.042	0.11	0.74	0.59	0.051	0.07	0.83

Linear regression analysis after application of a log10 transformation to the CSF biomarker measurements and an inverse rank normal transformation for IgG index, OCB count and WBC count at diagnostic lumbar puncture as outcome variables. Age at diagnosis and sex were included as covariates, with addition of the rs150192398 genotype for CHIT1 (available for 150 out of 180 CHIT1 measurements). β represents the regression coefficient. Uncorrected p-values (*P*) are shown and $P \leq 0.05$ are indicated in bold. IgG, immunoglobulin G; OCB, oligoclonal bands; WBC, white blood cells. n = number of measurements included in linear regression.

Supplementary Table 4. Simple linear regression of CSF biomarkers at diagnosis with single-time-point disease activity parameters at follow-up.

Parameter	Outcome	Variable (Outcome)	CHIT1 (n = 180)		CHI3L1 (n = 135)		sTREM2 (n = 118)		GPNMB (n = 192)		CCL18 (n = 187)	
			**β**	***P***	**β**	***P***	**β**	***P***	**β**	***P***	**β**	***P***
Disability	Primary	ARMSS	0.65	**8.86×10^{-5} [a]**	1.04	0.013[b]	-0.31	0.40	0.69	0.046[b]	0.78	0.021[b]
	Secondary	MSSS	0.59	**9.35×10^{-5} [a]**	0.85	0.026[b]	-0.20	0.53	0.53	0.10	0.70	0.030[b]
	Secondary	EDSS	0.51	**9.16×10^{-5} [a]**	0.88	0.0054[b]	-0.06	0.84	0.49	0.078	0.58	0.034[b]
Relapse	Primary	ARR, untreated	0.06	0.67	0.56	0.073	-0.17	0.55	-0.09	0.73	0.23	0.41

Linear regression analysis after application of a log10 transformation to the CSF biomarker measurements and an inverse rank normal transformation for ARMSS, MSSS, EDSS and untreated ARR as outcome variables. For ARMSS, MSSS and EDSS, the most recent assessment for each MS patient was used. Primary and secondary outcomes are indicated. Age at diagnosis and sex were included as covariates, with addition of the rs150192398 genotype for CHIT1 (available for 150 out of 180 CHIT1 measurements). β represents the regression coefficient. Bonferroni correction for multiple testing (five independent biomarkers and four outcome variables) resulted in a significance threshold of ≤ 0.0025. [a]Corrected p-values (*P*) ≤ 0.0025 are indicated in bold. [b]Uncorrected $P \leq 0.05$. ARMSS, Age-Related Multiple Sclerosis Severity; MSSS, Multiple Sclerosis Severity Score; EDSS, Expanded Disability Status Scale; ARR, annualized relapse rate. n = number of measurements included in linear regression.

Supplementary Table 5. Multiple linear regression of CSF biomarkers at diagnosis with single-time-point disease activity parameters at follow-up.

			CHIT1 (n = 180)		GPNMB (n = 192)		CCL18 (n = 187)	
**Parameter**	**Outcome**	**Variable (Outcome)**	**β**	***P***	**β**	***P***	**β**	***P***
Disability	Primary	ARMSS	0.58	**0.0011**	0.28	0.55	0.61	0.12
	Secondary	MSSS	0.55	**0.00085**	0.31	0.46	0.60	0.095
	Secondary	EDSS	0.50	**0.00057**	0.19	0.61	0.35	0.27
Relapse	Primary	ARR, untreated	0.09	0.56	-0.11	0.79	0.15	0.65

Multiple linear regression analysis with CHIT1, GPNMB and CCL18 combined as covariates. CHI3L1 and NfL have already been addressed in our previous work[1]. Since sTREM2 was not nominally significant ($P \leq 0.05$) with any of the outcome variables in Table S4, sTREM2 was omitted. A log10 transformation to the CSF biomarker measurements and an inverse rank normal transformation for ARMSS, MSSS, EDSS and untreated ARR as outcome variables was applied. For ARMSS, MSSS and EDSS, the most recent assessment for each MS patient was used. Primary and secondary outcomes are indicated. Age at diagnosis and sex were included as covariates, with addition of the rs150192398 genotype for CHIT1 (available for 150 out of 180 CHIT1 measurements). β represents the regression coefficient. Significant p-values (*P*) after Bonferroni correction for multiple testing ($P \leq 0.0042$) are indicated in bold. ARR, annualized relapse rate. n = number of measurements included in linear regression.

Supplementary Table 6. Mixed-effects linear regression models of CSF CHIT1 at diagnosis with multi-time-point EDSS assessments at follow-up.

	**Model 1** R^2 = 0.31, AIC = 636.1, BIC = 662.9					**Model 2** R^2 = 0.30, AIC = 628.4, BIC = 662.9					**Model 3** R^2 = 0.32, AIC = 620.5, BIC = 658.8				
	Parameter estimates					***Parameter estimates***					***Parameter estimates***				
Fixed effects	*β*	*CI*	*SE*	*t*	*P*	*β*	*CI*	*SE*	*t*	*P*	*β*	*CI*	*SE*	*t*	*P*
CHIT1	0.24	0.12; 0.36	0.06	3.97	**0.00011**	0.21	0.09; 0.34	0.06	3.60	**0.00045**	0.27	0.15; 0.39	0.06	4.34	**2.63×10^{-5}**
Time from diagnosis to EDSS	0.18	0.12; 0.23	0.03	6.77	**1.13×10^{-10}**	0.20	0.13; 0.27	0.03	5.86	**2.71×10^{-7}**	0.20	0.14; 0.27	0.03	6.55	**5.56×10^{-8}**
Age at diagnosis	0.43	0.31; 0.54	0.06	7.38	**8.01×10^{-12}**	0.43	0.31; 0.54	0.06	7.39	**8.15×10^{-12}**	0.42	0.31; 0.54	0.06	7.25	**1.72×10^{-11}**
Sex: Male	0.18	-0.06; 0.42	0.12	1.50	0.14	0.20	-0.04; 0.43	0.12	1.64	0.10	0.18	-0.05; 0.42	0.12	1.53	0.13
CHIT1*Time	-	-	-	-	-	-	-	-	-	-	0.11	0.04; 0.17	0.03	3.28	**0.0019**

Random effects	*Variance*	*SD*	*Correlation*	*Variance*	*SD*	*Correlation*	*Variance*	*SD*	*Correlation*
Patient	0.45	0.67	-	0.48	0.70	0.27	0.49	0.70	0.30
Time from diagnosis to EDSS	-	-	-	0.05	0.21		0.03	0.18	
Residual	0.15	0.39	-	0.11	0.32	-	0.11	0.33	-

Comparison of three mixed-effects linear regression models that assess the correlation between CSF CHIT1 at diagnosis and multi-time-point EDSS assessments. Model 1: contains a random intercept to allow for variance in EDSS scores across MS patients. Model 2: contains the same random intercept as model 1, but also a random slope to allow for variance in EDSS scores across time. Model 3: contains the same random effects as model 2, but also an interaction effect between CHIT1 and time from diagnosis to EDSS assessment. For sex, female is the reference group. All mixed-effects models were conducted on 157 MS patients with a total of 340 EDSS entries. A log10 transformation to the CHIT1 measurements and an inverse rank normal transformation for EDSS as outcome variable was applied. CHIT1, time from diagnosis to EDSS and age at diagnosis values were scaled. Model 3 displayed the best fit with a > 15-unit drop in Akaike information criterion (AIC; as an estimator of prediction error) and reductions in residual error as the models were tailored. Likelihood ratio tests were performed; χ^2 model 1 *versus* model 2 = 11.62 with p-value (*P*) = 0.0029. χ^2 model 2 *versus* model 3 = 9.94 with *P* = 0.0016. – indicates variables that were not included in the model. β, regression coefficient; BIC, Bayesian information criterion; CI, 95% confidence interval; R^2, R^2 of fixed effects; SD, standard deviation; SE, standard error; t, t-statistic.

Supplementary Table 7. EDSS assessments for multi-time-point analysis.

	Number of EDSS assessments				
	≥ 1	≥ 2	≥ 3	≥ 4	≥ 5
n (%)	157 (81.8%)	104 (54.2%)	53 (27.6%)	23 (12.0%)	3 (1.6%)
EDSS, median (IQR)	1.0 (0.0 - 2.5)	1.8 (1.0 - 3.5)	2.0 (1.0 - 3.5)	2.0 (1.0 - 6.3)	6.0 (4.0 - 6.8)
Time from diagnosis to EDSS, years, median (IQR)	1.5 (0.7 - 3.0)	5.0 (3.3 - 7.2)	7.5 (5.4 - 9.4)	9.3 (7.6 - 11.5)	11.4 (9.8 - 12.9)

Number of MS patients in function of the number of EDSS assessments. A total of 157 out of 192 analyzed MS patients had data for variables CHIT1, time from diagnosis to EDSS, age at diagnosis and sex in the mixed-effects model as well as at least 1 EDSS assessment. IQR, interquartile range.

Supplementary Table 8. Epidemiological data of neuropathology patients.

MS case	Type of MS	Disease duration (months)	Nr. active lesions (CHIT1+)	% CHIT1+ active lesions	Nr. inactive lesions (CHIT1+)	% CHIT1+ inactive lesions
#1	AMMS	0.2	1(1)	100	0(0)	NA
#2	AMMS	1.5	1(1)	100	0(0)	NA
#3	AMMS	1.5	3(3)	100	0(0)	NA
#4	AMMS	5	5(5)	100	0(0)	NA
#5	AMMS	2	1(1)	100	0(0)	NA
#6	RRMS	30	4(4)	100	2(0)	0
#7	PPMS	204	1*(0)	0	0(0)	NA
#8	PPMS	61	2(2)	100	0(0)	NA
#9	PPMS	87	0(0)	NA	2(0)	0
#10	SPMS	137	1(1)	100	0(0)	NA
#11	SPMS	410	1(1)	100	3(0)	0
#12	SPMS	263	1*(1)	100	1(0)	0
**Total**			**21(20)**	**95.2**	**8(0)**	**0**

MS cases used for neuropathological evaluation of CHIT1. Types of MS: AMMS (acute monophasic MS), RRMS (relapsing-remitting MS), SPMS (secondary progressive MS) and PPMS (primary progressive MS). Median age: 46.5 years old (range: 34-68). Sex ratio (female:male): 3:9. * = smoldering lesion.

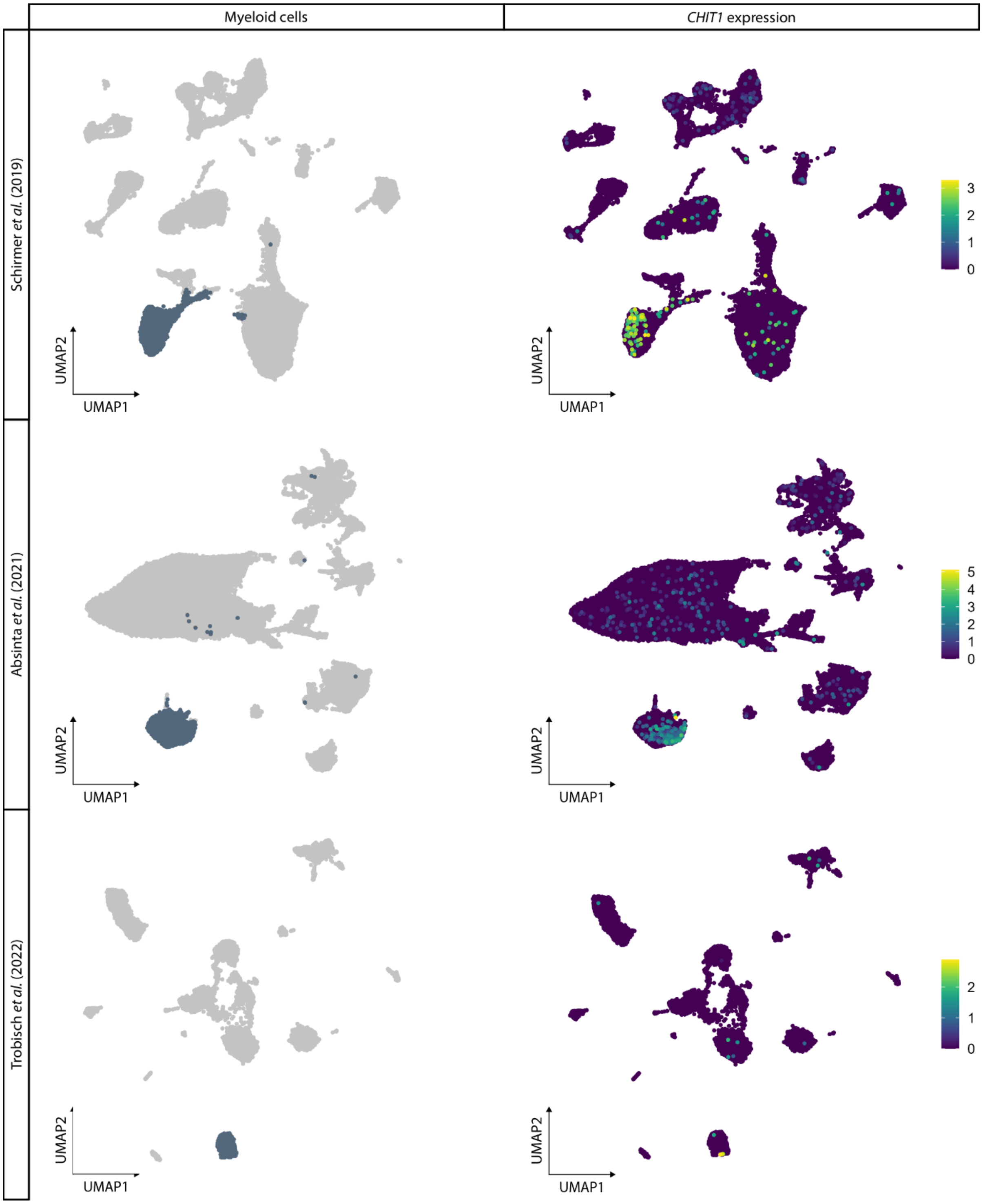

Supplementary Fig. 1. CNS cell type-specificity of *CHIT1* expression. UMAP plots depict general clustering of CNS cells from the Schirmer *et al.*[2], Absinta *et al.*[3] and Trobisch *et al.*[4] datasets. Plots on the left show myeloid cells defined as cells co-expressing *PTPRC*, *CSF1R*, *P2RY12* and *ITGAM*. Plots on the right show log-normalized *CHIT1* expression. The dataset from Miedema *et al.*[5] is not shown as it consisted of only FACS-sorted myeloid cells.

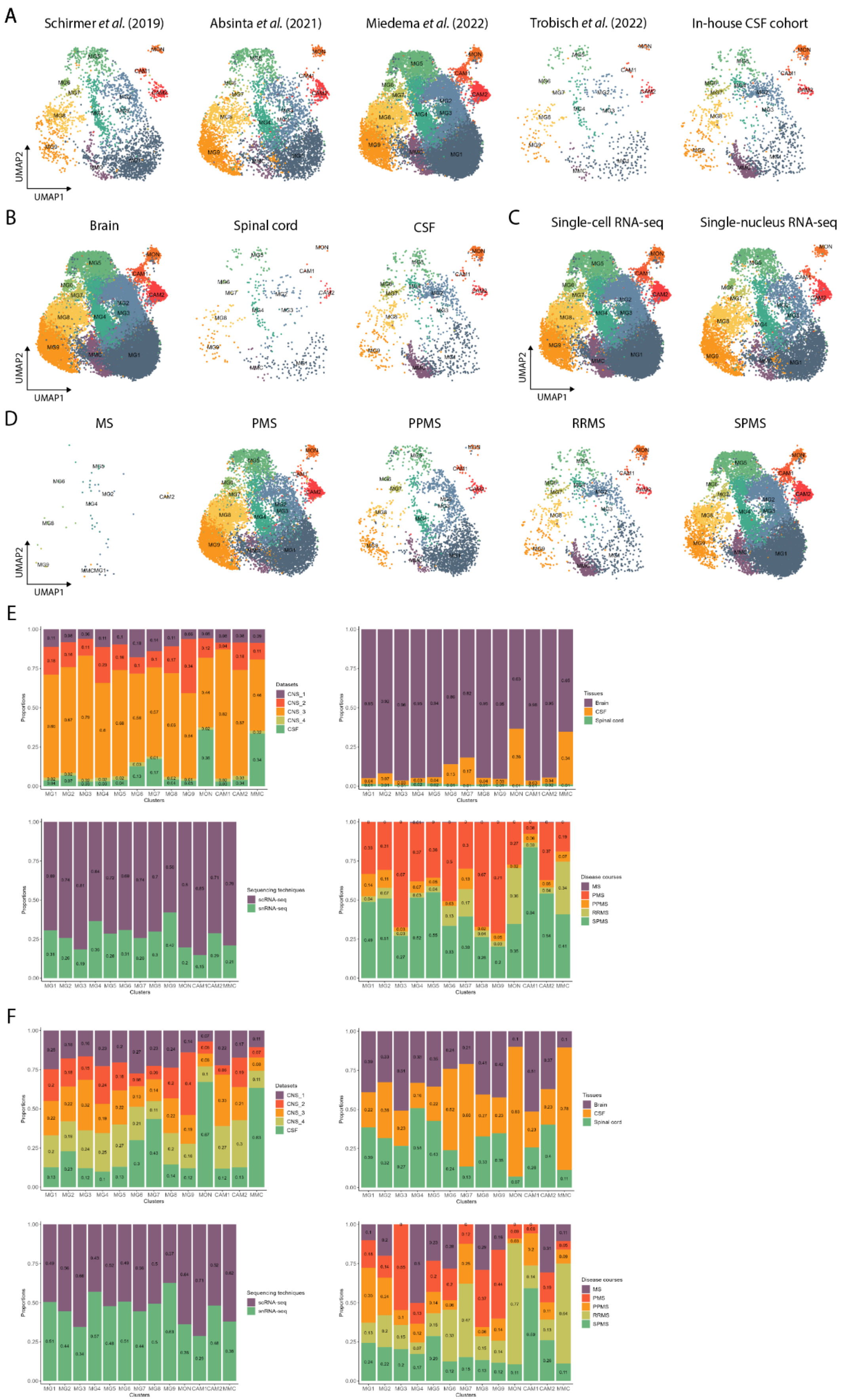
A
Schirmer et al. (2019)
Absinta et al. (2021)
Miedema et al. (2022)
Trobisch et al. (2022)
In-house CSF cohort
UMAP2
UMAP1
B
Brain
Spinal cord
CSF
C
Single-cell RNA-seq
Single-nucleus RNA-seq
D
MS
PMS
PPMS
RRMS
SPMS
E
F
Datasets
CNS_1
CNS_2
CNS_3
CNS_4
CSF
Tissues
Brain
CSF
Spinal cord
Sequencing techniques
scRNA-seq
snRNA-seq
Disease courses
MS
PMS
PPMS
RRMS
SPMS
Proportions
Clusters
MG1
MG2
MG3
MG4
MG5
MG6
MG7
MG8
MG9
MON
CAM1
CAM2
MMC

Supplementary Fig. 2. Evaluation of myeloid cell integration from all five datasets. Myeloid cells from all samples of the five datasets (our in-house CSF dataset and four previously published CNS datasets[2, 3, 4, 5]) were integrated using the Harmony v0.1.1 package[6] with default parameters. Each individual sample was treated as a batch and sequencing technique (scRNA-seq or snRNA-seq) was included as a covariate. UMAP plots show the myeloid subclustering of the integrated dataset, split by original dataset (**A**), tissue of origin (**B**), sequencing technique (**C**) and MS subtype (**D**). Stacked bar plots show the absolute cell distribution (**E**) or relative cell distribution weighed to the total number of cells (**F**) of each original dataset, tissue of origin, sequencing technique and MS subtype over all clusters. CSF, cerebrospinal fluid; MS, multiple sclerosis; PMS, progressive MS; PPMS, primary progressive MS; RRMS, relapsing-remitting MS; SPMS, secondary progressive MS; scRNA-seq, single-cell RNA-seq; snRNA-seq, single-nucleus RNA-seq. Source data are provided as a Source Data file.

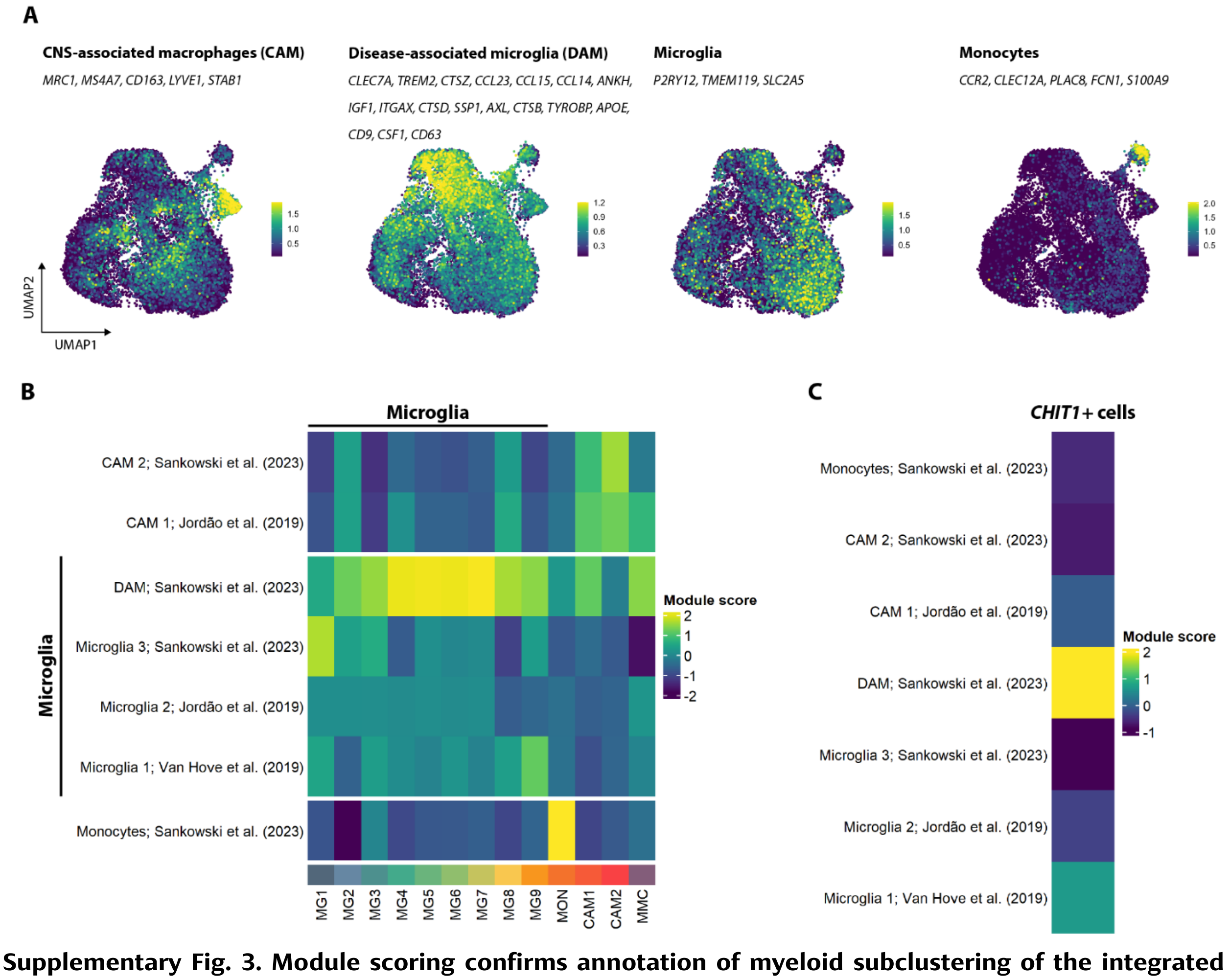

Supplementary Fig. 3. Module scoring confirms annotation of myeloid subclustering of the integrated dataset. (**A**) UMAP plots show the module scores for myeloid cell types as defined by Sankowski *et al.*[7] across our myeloid subclustering. Heatmaps depict the module scores per cluster (**B**) and across all *CHIT1*+ cells (**C**) for microglia, CNS-associated macrophages and monocytes from three recent reference works[7, 8, 9]. Source data are provided as a Source Data file.

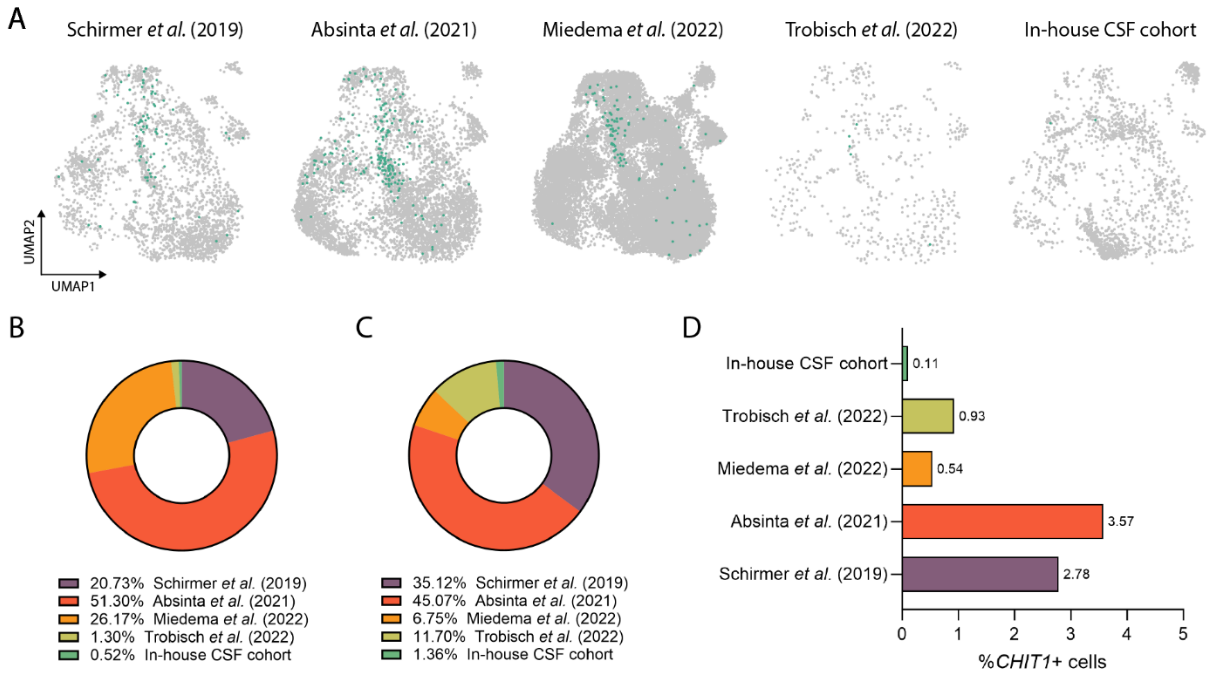

Supplementary Fig. 4. Distribution of *CHIT1*+ cells over individual datasets. (**A**) UMAP plots show the myeloid subclustering of the integrated dataset, split by original dataset. Cells expressing *CHIT1* are indicated in green. Donut charts depict the absolute cell distribution (**B**) or relative cell distribution weighed to the total number of cells (**C**) of each original dataset to the pool of *CHIT1*+ cells. (**D**) Bar plot shows the percentage of *CHIT1*+ cells in each original dataset. CSF, cerebrospinal fluid. Source data are provided as a Source Data file.

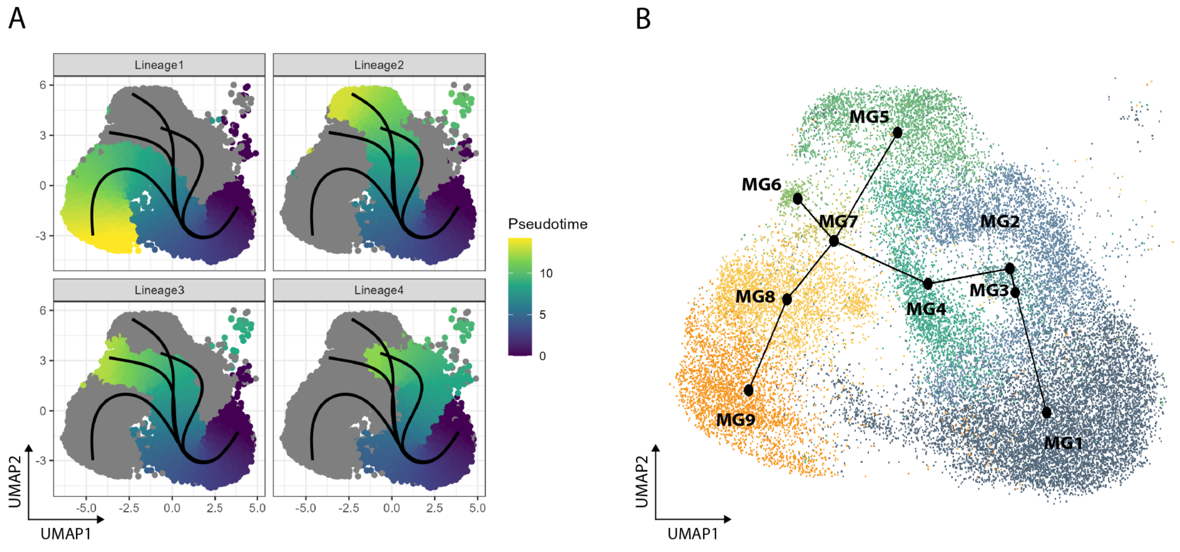

Supplementary Fig. 5. Pseudotime trajectory analysis on clusters MG1-9. (**A**) UMAP plots show the four lineages identified in the microglia clusters (MG1-9). Color scale depicts the pseudotime value of the cells. (**B**) UMAP plot shows the microglia clusters with the global lineage structure and branching points. Lineage 1: MG1, MG2, MG4, MG7, MG8, MG9. Lineage 2: MG1, MG2, MG4, MG7, MG5. Lineage 3: MG1, MG2, MG4, MG7, MG6. Lineage 4: MG1, MG2, MG3.

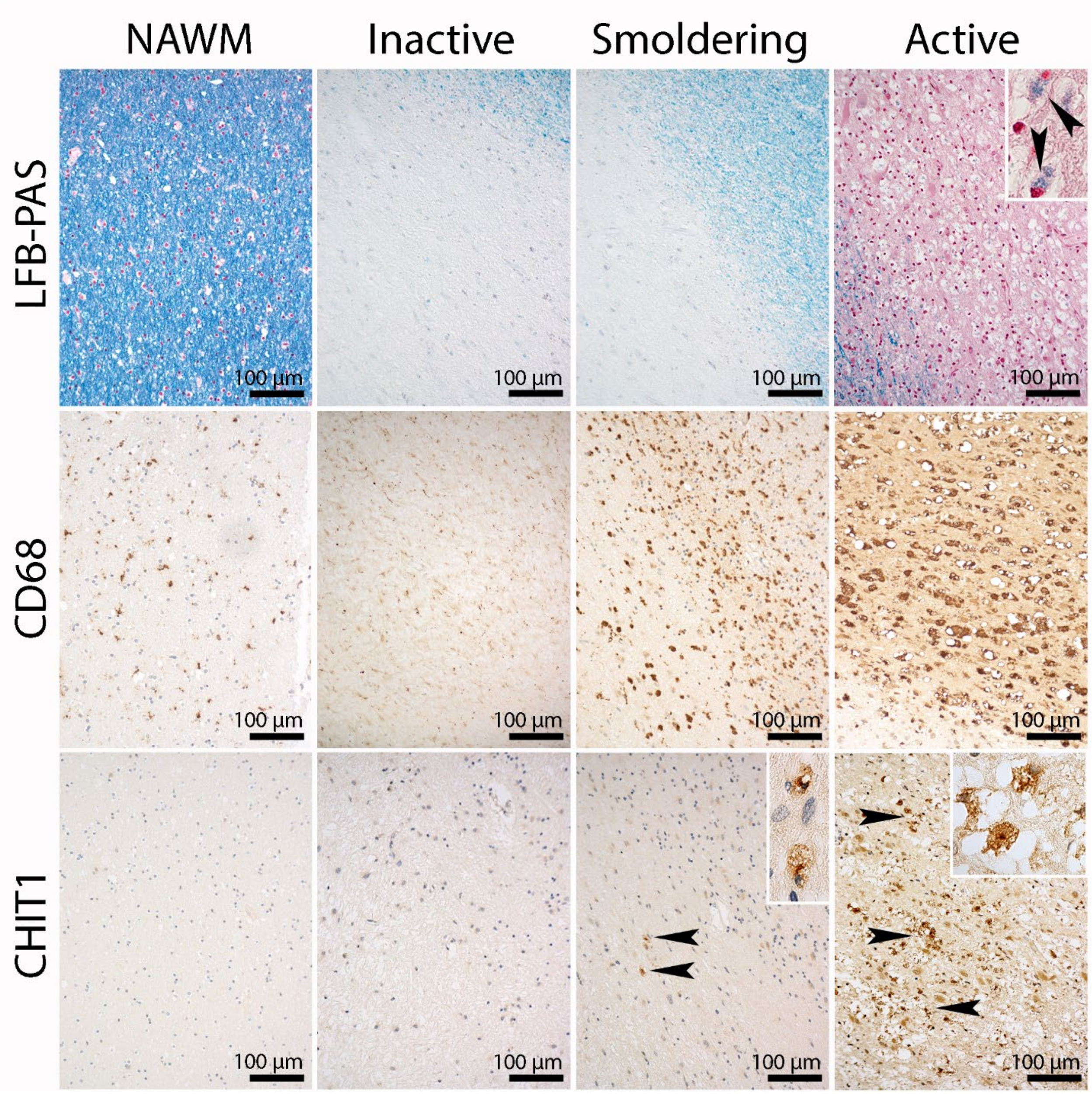

Supplementary Fig. 6. CHIT1-reactivity in MS lesions. Representative images of staining for Luxol Fast Blue-Periodic acid Schiff (LFB-PAS), CD68 and CHIT1 in normal-appearing white matter (NAWM) and different lesion types for all 12 MS patients. In NAWM, CD68 staining shows activation of microglia/macrophages, but CHIT1+ cells are absent. In an inactive lesion, the LFB-PAS staining shows the absence of myelin. CD68 in these lesions again shows reactivity, but larger, lipid-laden microglia/macrophages are absent and also CHIT1 immunoreactivity is absent. In smoldering lesions, the lesion edge shows enhanced CD68 staining in microglia/macrophages on the edge of the lesion. CHIT1-immunoreactivity is limited to few lipid-laden cells (arrowheads, higher magnification in inset). The active lesion on the right of the figure in the LFB-PAS staining shows absence of myelin but the presence of early LFB+ degradation products in microglia/macrophages (arrowheads in inset). These larger phagocytic microglia/macrophages can also be appreciated in the CD68 staining. Unlike in inactive lesions, CHIT1 immunoreactivity is seen in many small and larger phagocytic cells (arrowheads, higher magnification in inset).

Supplementary references

1. Oldoni, E. *et al.* CHIT1 at Diagnosis Reflects Long-Term Multiple Sclerosis Disease Activity. *Ann. Neurol.* **87**, 633-645 (2020).

2. Schirmer, L. *et al.* Neuronal vulnerability and multilineage diversity in multiple sclerosis. *Nature* **573**, 75-82 (2019).

3. Absinta, M. *et al.* A lymphocyte–microglia–astrocyte axis in chronic active multiple sclerosis. *Nature* **597**, 709-714 (2021).

4. Trobisch, T. *et al.* Cross-regional homeostatic and reactive glial signatures in multiple sclerosis. *Acta Neuropathol.* **144**, 987-1003 (2022).

5. Miedema, A. *et al.* Brain macrophages acquire distinct transcriptomes in multiple sclerosis lesions and normal appearing white matter. *Acta Neuropathol. Commun.* **10**, 8 (2022).

6. Korsunsky, I. *et al.* Fast, sensitive and accurate integration of single-cell data with Harmony. *Nat. Methods* **16**, 1289-1296 (2019).

7. Sankowski, R. *et al.* Multiomic spatial landscape of innate immune cells at human central nervous system borders. *Nat. Med.* **30,** 186-198 (2023).

8. Jordão, M.J.C. *et al.* Single-cell profiling identifies myeloid cell subsets with distinct fates during neuroinflammation. *Science* **363**, eaat7554 (2019).

9. Van Hove, H. *et al.* A single-cell atlas of mouse brain macrophages reveals unique transcriptional identities shaped by ontogeny and tissue environment. *Nat. Neurosci.* **22**, 1021-1035 (2019).
