# Supplementary material for: CHIT1 at diagnosis predicts faster disability progression and reflects early microglial activation in multiple sclerosis
Source: Nat Commun. 2024 Jun 12;15:5013. doi: 10.1038/s41467-024-49312-y (PMC11169395; doi:10.1038/s41467-024-49312-y)
Supplement: Supplementary file 3 — Description of Additional Supplementary Files [file 41467_2024_49312_MOESM3_ESM.pdf]

Description of Additional Supplementary Files

File Name: Supplementary Data 1
Description: Raw biomarker measurements.

File Name: Supplementary Data 2
Description: Description of all included scRNA-seq and snRNA-seq datasets.

File Name: Supplementary Data 3
Description: Canonical marker genes and gene modules used for annotation and module scoring analyses.

File Name: Supplementary Data 4
Description: Differentially expressed genes across all myeloid clusters.

File Name: Supplementary Data 5
Description: Differentially expressed genes CHIT1+ versus CHIT1- cells.

File Name: Supplementary Data 6
Description: Differential gene expression analyses across pseudotime trajectories.
