# Supplementary material for: CHIT1 at diagnosis predicts faster disability progression and reflects early microglial activation in multiple sclerosis
Source: Nat Commun. 2024 Jun 12;15:5013. doi: 10.1038/s41467-024-49312-y (PMC11169395; doi:10.1038/s41467-024-49312-y)
Supplement: Supplementary file 10 — Reporting Summary [file 41467_2024_49312_MOESM10_ESM.pdf]

nature portfolio

Corresponding author(s): Bénédicte Dubois

Last updated by author(s): May 21, 2024

Reporting Summary

Nature Portfolio wishes to improve the reproducibility of the work that we publish. This form provides structure for consistency and transparency in reporting. For further information on Nature Portfolio policies, see our Editorial Policies and the Editorial Policy Checklist.

Statistics

For all statistical analyses, confirm that the following items are present in the figure legend, table legend, main text, or Methods section.

n/a	Confirmed	
☐	☒	The exact sample size (*n*) for each experimental group/condition, given as a discrete number and unit of measurement
☐	☒	A statement on whether measurements were taken from distinct samples or whether the same sample was measured repeatedly
☐	☒	The statistical test(s) used AND whether they are one- or two-sided<br>*Only common tests should be described solely by name; describe more complex techniques in the Methods section.*
☐	☒	A description of all covariates tested
☐	☒	A description of any assumptions or corrections, such as tests of normality and adjustment for multiple comparisons
☐	☒	A full description of the statistical parameters including central tendency (e.g. means) or other basic estimates (e.g. regression coefficient) AND variation (e.g. standard deviation) or associated estimates of uncertainty (e.g. confidence intervals)
☐	☒	For null hypothesis testing, the test statistic (e.g. *F*, *t*, *r*) with confidence intervals, effect sizes, degrees of freedom and *P* value noted<br>*Give P values as exact values whenever suitable.*
☒	☐	For Bayesian analysis, information on the choice of priors and Markov chain Monte Carlo settings
☐	☒	For hierarchical and complex designs, identification of the appropriate level for tests and full reporting of outcomes
☐	☒	Estimates of effect sizes (e.g. Cohen's *d*, Pearson's *r*), indicating how they were calculated

Our web collection on statistics for biologists contains articles on many of the points above.

Software and code

Policy information about availability of computer code

Data collection	All previously published CNS datasets were downloaded as FASTQ files from the Sequence Read Archive (SRA) under BioProject accession numbers PRJNA544731, PRJNA749443, PRJNA743676 and PRJNA726991. SRA-Toolkit version 3.0.0 was used.
Data analysis	We used Cell Ranger version 7.1.0 (10x Genomics) software to process all the single-cell/nucleus datasets. Further analysis was done via bioinformatics pipeline in R v4.2.2 which incorporated several R packages: SoupX v1.6.2, scDblFinder v1.12.0, Seurat v4.3.0, SCTransform v0.3.5, Harmony v0.1.1, clustree v0.5.0, SingleR v2.0.0, celldex v1.8.0, clusterProfiler v4.6.2, slingshot v2.6.0 and tradeSeq v1.12.0. We performed single-time-point analysis and mixed-effects models in R v4.0.1 and used lme4 v1.1-31, afex v1.2-1 and ggplot v3.4.2. Bar plot in Fig. 4C was created with GraphPad Prism v10.0.0. A custom machine learning model as described in D'hondt et al. (Artificial Intelligence in Medicine., 2023) was developed Python 3.8.10. The code scripts to recreate the main figures and results are accessible in Zenodo via GitHub under the DOI 10.5281/zenodo.11235175 [https://zenodo.org/doi/10.5281/zenodo.11235175]. No unpublished, standalone software/tool/program/package was built.

For manuscripts utilizing custom algorithms or software that are central to the research but not yet described in published literature, software must be made available to editors and reviewers. We strongly encourage code deposition in a community repository (e.g. GitHub). See the Nature Portfolio guidelines for submitting code & software for further information.

Data

Policy information about availability of data

All manuscripts must include a data availability statement. This statement should provide the following information, where applicable:

- Accession codes, unique identifiers, or web links for publicly available datasets
- A description of any restrictions on data availability
- For clinical datasets or third party data, please ensure that the statement adheres to our policy

All data that support the main findings in this study are available in the manuscript or the supplementary materials. The previously published CNS scRNA-seq and snRNA-seq datasets were downloaded from the Sequence Read Archive (SRA) under BioProject accession numbers PRJNA544731, PRJNA749443, PRJNA743676 and PRJNA726991. Our previously unpublished CSF scRNA-seq cohort has been uploaded to the SRA under BioProject accession number PRJNA996357. Raw biomarker measurements, processed sc/snRNA-seq data (e.g. differential gene expression), technical sc/snRNA-seq information and details of the included patients are provided in Supplementary Data 1-6.

Research involving human participants, their data, or biological material

Policy information about studies with human participants or human data. See also policy information about sex, gender (identity/presentation), and sexual orientation and race, ethnicity and racism.

Reporting on sex and gender	The term “sex” was used to indicate a biological attribute. Information about sex was collected from medical records (as on the identity cards). Since sex may influence MS severity, we included sex as a covariate in the biomarker analysis (single- and multi-time-point analyses and machine learning models) as described in the methods, results and/or legends. However, this study was not designed with the aim to demonstrate differences between sexes. Disaggregated data based on sex is not available in the source data or supplementary materials.
Reporting on race, ethnicity, or other socially relevant groupings	Since ethnicity may influence MS severity, ethnicity was incorporated among many other variables as a covariate in the machine learning models. Ethnicity was collected from medical records. Ethnicity was not used as a proxy for other variables. This study was not designed with the aim to demonstrate differences between ethnicities.
Population characteristics	Age at diagnosis (LP), sex and the rs150192398 genotype for CHIT1 were included as subject-relevant covariates in the single-time-point analyses. CHIT1 concentration, time from diagnosis (LP) to EDSS, age at diagnosis (LP) and sex were included as subject-relevant covariates in the mixed-effects models. Disease course, EDSS at diagnosis (LP), age at diagnosis (LP), disease duration at LP, CHIT1 concentration, year of diagnosis, OCB count, IgG index, CHI3L1 concentration, age at disease onset, WBC count, albumin levels, sTREM2 concentration, OCB status, ethnicity, sex, NfL concentration, CCL18 concentration and GPNMB concentration were obtained from medical records or as described in the materials and methods. All MS patients included in the CSF scRNA-seq analysis and biomarker analysis were untreated at sample collection.
Recruitment	All MS patients were recruited at the department of Neurology of the University Hospitals Leuven (Belgium) by the same expert neurologist (BD). MS patients were recruited independent of subject or disease characteristics. Post-mortem brain samples were collected from the archives of the Center for Brain Research at the Medical University of Vienna by the same expert pathologist (JBa).
Ethics oversight	Ethics Committee of the University Hospitals Leuven and Ethics Committee of the Medical University of Vienna.

Note that full information on the approval of the study protocol must also be provided in the manuscript.

Field-specific reporting

Please select the one below that is the best fit for your research. If you are not sure, read the appropriate sections before making your selection.

☒ Life sciences ☐ Behavioural & social sciences ☐ Ecological, evolutionary & environmental sciences

For a reference copy of the document with all sections, see nature.com/documents/nr-reporting-summary-flat.pdf

Life sciences study design

All studies must disclose on these points even when the disclosure is negative.

Sample size	Sample size biomarker analysis = 196 MS patients. After quality control, 192 MS patients were included in the single-time-point analysis and 157 MS patients were included in the multi-time-point analyses and machine learning models. Sample size single-cell/nucleus transcriptomics = 37 MS patients. Single-cell/nucleus data of 26 MS patients was collected from publicly available CNS datasets. Single-cell/nucleus data of 11 MS patients was collected from our in-house CSF cohort. Sample size neuropathological evaluation = 12 MS patients (21 active lesions and 8 inactive lesions). No sample-size calculations were performed.
Data exclusions	With regard to the biomarker analysis, samples with a coefficient of variance more than 20% or CSF biomarker concentrations below the detection limit were excluded. These exclusion criteria were pre-established. Samples above the upper detection limit were remeasured using a different dilution factor where possible or were eliminated otherwise. For the sc/snRNA-seq analysis, data was excluded as described in the quality control paragraph of the methods. These thresholds for quality control are in line with the current literature for single-cell/nucleus transcriptomics.

Replication	With regard to the biomarker analysis, CSF protein concentrations were measured in duplicate and blinded to clinical data. Mean values across duplicates were used for analysis. Machine learning models, independent of single- and multi-time-point analyses, replicated that CHIT1 is the most robust predictor for disability progression. For the sc/snRNA-seq analysis, the unsupervised/unbiased sc/snRNA-seq technique was applied to generate an initial hypothesis. Afterwards, the most important results were replicated at the protein level with neuropathological evaluation on post-mortem brain samples.
Randomization	We allocated patients to the MS group in accordance with the 2017 revised McDonald criteria.
Blinding	CSF protein concentrations were measured in duplicate and blinded to clinical data. We performed the single- and multi-time-point analyses and machine learning models blinded for disability progression. EDSS scores were used as input for the models, but a distinction between fast and slow progressors was never predefined.

Reporting for specific materials, systems and methods

We require information from authors about some types of materials, experimental systems and methods used in many studies. Here, indicate whether each material, system or method listed is relevant to your study. If you are not sure if a list item applies to your research, read the appropriate section before selecting a response.

Materials & experimental systems

n/a	Involved in the study	
☐	☒	Antibodies
☒	☐	Eukaryotic cell lines
☒	☐	Palaeontology and archaeology
☒	☐	Animals and other organisms
☐	☒	Clinical data
☒	☐	Dual use research of concern
☒	☐	Plants

Methods

n/a	Involved in the study	
☒	☐	ChIP-seq
☒	☐	Flow cytometry
☒	☐	MRI-based neuroimaging

Antibodies

Antibodies used	Immune light microscopical staining: primary antibodies CHIT1 (#HPA010575, Sigma) and CD68 (#M0814, Dakocytomation). Secondary biotin anti-rabbit-conjugated antibody (#711-065-152, Jackson) or biotin-anti-mouse-conjugated antibody (Jackson #715-065-150).<br>Immunofluorescent multiplex labeling: primary antibodies CHIT1 (#HPA010575, Sigma), Iba1 (#019-19741, Wako), PLP (#MCA839G, Bio-Rad), GFAP (#MS-1376, Thermo Scientific), TMEM119 (#HPA051870, Sigma) and CD68 (#M0814, Dakocytomation). Secondary Opal Polymer horseradish peroxidase conjugated antibodies (#ARH1001EA, Akoya).
Validation	All antibodies used are commercially available and validated by the manufacturer.

Clinical data

Policy information about clinical studies
All manuscripts should comply with the ICMJEguidelines for publication of clinical research and a completedCONSORT checklist must be included with all submissions.

Clinical trial registration	No clinical trial.
Study protocol	No clinical trial.
Data collection	No clinical trial.
Outcomes	No clinical trial.

Plants

Seed stocks

Report on the source of all seed stocks or other plant material used. If applicable, state the seed stock centre and catalogue number. If plant specimens were collected from the field, describe the collection location, date and sampling procedures.

Novel plant genotypes

Describe the methods by which all novel plant genotypes were produced. This includes those generated by transgenic approaches, gene editing, chemical/radiation-based mutagenesis and hybridization. For transgenic lines, describe the transformation method, the number of independent lines analyzed and the generation upon which experiments were performed. For gene-edited lines, describe the editor used, the endogenous sequence targeted for editing, the targeting guide RNA sequence (if applicable) and how the editor was applied.

Authentication

Describe any authentication procedures for each seed stock used or novel genotype generated. Describe any experiments used to assess the effect of a mutation and, where applicable, how potential secondary effects (e.g. second site T-DNA insertions, mosiacism, off-target gene editing) were examined.
